# Differential effects of bilateral hippocampal CA3 damage on the implicit learning and recognition of complex event sequences

**DOI:** 10.1080/17588928.2024.2315818

**Published:** 2024-02-21

**Authors:** Thomas D. Miller, Christopher Kennard, Penny A. Gowland, Chrystalina A. Antoniades, Clive R. Rosenthal

**Affiliations:** a Wellcome Centre for Human Neuroimaging, University College London, London, UK; b National Hospital for Neurology and Neurosurgery, Queen Square, London, UK; c Nuffield Department of Clinical Neurosciences, University of Oxford, Oxford, UK; d Sir Peter Mansfield Imaging Centre, School of Physics and Astronomy, University of Nottingham, Nottingham, UK

**Keywords:** Amnesia, sequence learning, implicit memory, recognition memory, item memory, hippocampal subfields, autoimmune encephalitis

## Abstract

Learning regularities in the environment is a fundament of human cognition, which is supported by a network of brain regions that include the hippocampus. In two experiments, we assessed the effects of selective bilateral damage to human hippocampal subregion CA3, which was associated with autobiographical episodic amnesia extending ~50 years prior to the damage, on the ability to recognize complex, deterministic event sequences presented either in a spatial or a non-spatial configuration. In contrast to findings from related paradigms, modalities, and homologue species, hippocampal damage did not preclude recognition memory for an event sequence studied and tested at four spatial locations, whereas recognition memory for an event sequence presented at a single location was at chance. In two additional experiments, recognition memory for novel single-items was intact, whereas the ability to recognize novel single-items in a different location from that presented at study was at chance. The results are at variance with a general role of the hippocampus in the learning and recognition of complex event sequences based on non-adjacent spatial and temporal dependencies. We discuss the impact of the results on established theoretical accounts of the hippocampal contributions to implicit sequence learning and episodic memory.

The medial temporal lobe (MTL), which includes the hippocampus, the adjacent entorhinal cortex, perirhinal cortex, and postrhinal cortex, supports episodic memory (Suzuki and Eichenbaum, 2000; Squire, 2009; Teyler & Rudy, 2007). Damage involving these brain regions is associated with impairments in memory for events, spatial order, and temporal order (DeVito & Eichenbaum, 2011; Eichenbaum et al., 2007; Kesner et al., 2002; Miller et al., 2017, 2020; O’Keefe & Nadel, 1978; Squire et al., 2004). Extensive work has characterized memory for spatial and temporal order as components of sequence learning that can be supported by a non-conscious, implicit process (Batterink et al., 2019; Knowlton & Squire, 1996;. Reber, 1967; Seger, 1994; Williams, 2020). Implicit sequence learning has been mapped onto a distributed cortico-subcortical network of regions that include the hippocampus (Albouy et al., 2006; Rosenthal et al., 2016; Schendan et al., 2003). Involvement of the hippocampus is perhaps surprising given its established role in episodic memory. However, the processing capabilities of the hippocampus are compatible with the computational demands associated with sequence learning (Hannula & Greene, 2012; Henke, 2010; Kim, 2019; Moscovitch et al., 2016), independently of whether there is conscious access to an event sequence (Rosenthal & Soto, 2016; Rosenthal et al., 2016; Soto et al., 2019). Importantly, the hippocampus is not an unitary anatomical structure, rather it is comprised of subregions with differing firing properties, connectivity, and function (Baker et al., 2016; Bartsch et al., 2011; Dalton et al., 2022; Kesner & Rolls, 2015; Miller et al., 2017, 2020). However, lesion studies of implicit sequence learning in humans have averaged across subregions (Curran, 1997; Reber & Squire, 1998; Shanks et al., 2006), largely because hippocampal damage seldom affects a single subfield or remains confined to the hippocampus (Baker et al., 2016; Bartsch et al., 2011; Miller et al., 2017, 2020; Wang et al., 2023).

The main aim of the current study was to test the contributions of the cornu ammonis 3 (CA3) subregion of the hippocampus proper (Amaral & Lavenex, 2007) – a region widely agreed to support episodic encoding and retrieval (Kesner & Rolls, 2015; Rebola et al., 2017) – to the implicit learning of complex, deterministic event sequences. We addressed this aim by examining the effects of selective bilateral damage to human CA3 on memory for implicit serial associations between events. All of the individuals with damage to CA3 were previously characterized using 7.0-Tesla functional and anatomical neuroimaging (Miller et al., 2017, 2020). Unlike prior lesion studies of the effects of hippocampal damage on implicit sequence learning, anatomical damage was confined to the hippocampus and involved a single etiology, autoimmune encephalitis (Figure 1). In line with prior evidence showing that CA3 lesions in rodents lead to little or no impact on recognition memory for novel objects and impaired episodic-like recall (de Souza Silva et al., 2016), our studies revealed that the mnemonic sequelae of selective damage to human CA3 were intact item recognition memory but impaired episodic remembering of personal events (McCormick et al., 2016a, 2016b, 2017, 2018; Miller et al., 2017, 2020; Spanò, Pizzamiglio, et al., 2020; Spanò, Weber, et al., 2020). Notably, the selective loss of personal events did not change across recent and remote memories up to ~50 years prior to CA3 damage (i.e., it was temporally ungraded/followed a flat gradient), which is broadly consistent with the results from human neuroimaging that indicates human CA3 supports recent and remote episodic retrieval (Bonnici & Maguire, 2017; Bonnici et al., 2013; Chadwick et al., 2014), but is at variance with recent results from comparative biological models in rodents (Atucha et al., 2023; Lux et al., 2016).

**Figure 1.**
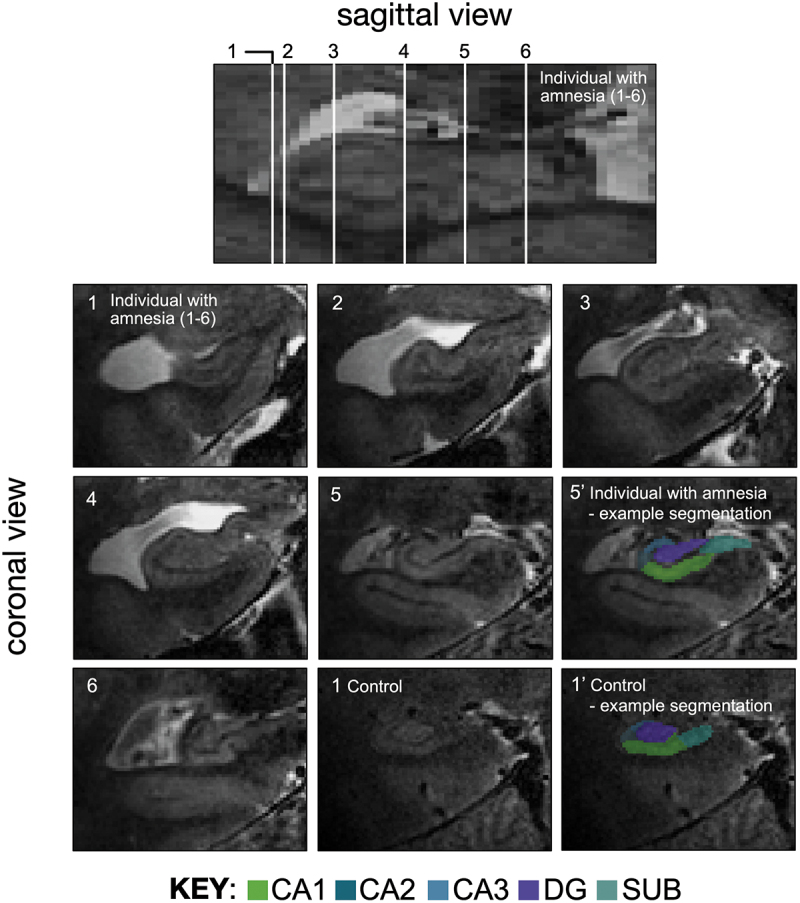
Location of the hippocampal damage was determined by manual segmentation of ultra high resolution anatomical magnetic resonance images, acquired in a previous study that reported results from 18 participants in the chronic phase of the LGI1-LE phenotype (Miller et al., 2017). Each white line (1–6) along the anterior – posterior axis on the sagittal view corresponds to the coronal views numbered 1–6 in an individual with amnesia, secondary to LGI1-limbic encephalitis. Manual segmentation of the subfields was performed on each coronal slice (52) along the full anterior-posterior hippocampal axis (Miller et al., 2017, 2020; Wisse et al., 2012). As an illustration of the topographic relationship between each of the segmented subfields, color shading on coronal slice 5’ provides an example of manual segmentation applied to slice 5 in the individual with amnesia. Coronal slice 1 in an example control participant corresponds to an anterior location that approximates to line 1 in the example individual with amnesia. Color shading on control coronal slice 1′ provides an example of the manual segmentation protocol applied to control slice 1 in the neuroimaging control participant. The color key under the 3-D fast spin-echo coronal images corresponds to CA1, CA2, CA3, DG (dentate gyrus), and SUB (subiculum) hippocampal subfields. Sagittal and coronal views based on 7.0-Tesla 3-D fast-spin echo T2-weighted fast spin-echo partial volumes, acquired at 0.39 × 0.39 × 1.0 mm^3^ spatial resolution in 52 contiguous oblique coronal sections (perpendicular to hippocampal axis). Adapted from (Miller et al., 2017), published under CC by license, http://creativecommons.org/licenses/by/4.0/.

Several approaches have been developed to assess the implicit learning of regularities in the environment (Chun & Phelps, 1999; Hopkins et al., 2004; Knowlton & Squire, 1996; Reber, 1967; Schapiro et al., 2014; Seger, 1994; Williams, 2020), and a key paradigm used in laboratory setting is the serial reaction time task (SRT task) (Cleeremans & McClelland, 1991; Hannula & Greene, 2012; Nissen & Bullemer, 1987; Rosenthal et al., 2009, 2013). In the standard version of the SRT task, participants are presented with a sequential series of visual targets that appear in one of four fixed screen locations aligned along the horizontal meridian of a computer screen. The onset of each target cues a response in the form of a manual key press mapped to one of four corresponding spatial locations. Implicit learning is inferred when information is acquired about the complex, deterministic sequence without conscious access either to what was learned or the fact that learning occurred (Cleeremans, 1993; Cohen et al., 1990; Frensch & Runger, 2003; Reber & Squire, 1994). It has been suggested that introducing a probabilistic component to an event sequence can minimize the level of awareness associated with the newly acquired knowledge (Rosenthal et al., 2009; Vandenberghe et al., 2006). However, the empirical foundation on which to base a lack of conscious awareness remains an open question (Barth et al., 2019; Cleeremans & Jimenez, 1998; Destrebecqz & Cleeremans, 2001; Hannula et al., 2023; Rosenthal & Soto, 2016, 2016; Rosenthal et al., 2009, 2013; Shanks & John, 1994; Shanks & Johnstone, 1999; Soto et al., 2019; Vadillo et al., 2016, 2022). Thus, when learning visible event sequences, we restrict the term implicit so that it refers to learning regularities under conditions where a participant is not oriented to the presence of the embedded regularities.

Evidence from human lesion studies that have examined the necessity of the human hippocampus for sequence learning does not readily align with the ample evidence from human fMRI studies that implicates the hippocampus (Albouy et al., 2006, 2012, 2013; Rosenthal et al., 2016; Schendan et al., 2003). Early studies suggested that learning on the SRT task was intact in individuals with amnesia, when the event sequences were based on frequency and predictive first-order probabilities (i.e., simple associations were predicted by learning adjacent stimuli) (Nissen & Bullemer, 1987; Nissen et al., 1989). These results have been extended to show that humans with MTL pathology and hippocampal damage can develop sensitivity not only to first-order regularities but also to second-order regularities, when assessed using an indirect reaction time-based measure (i.e., a manual reaction time (RT) advantage for the trained sequence regularities vs a new, previously unseen sequence) (Curran, 1997; Reber & Squire, 1994; Shanks et al., 2006). However, in the study by Curran (1997), learning deterministic, second-order regularities was impaired on the indirect test when compared against control group performance and subsequent recognition memory was at chance in the amnesic and control groups. A notable feature of an event sequence based on second-order regularities is that no single location is sufficient to predict a forthcoming location; instead, a location can be predicted only with the knowledge of two proceeding locations (i.e., the sequence was based on a higher-order (complex) second-order conditional generation rule rather than on simple adjacent dependencies; J. Reed and Johnson (1994)). In a later study, sensitivity to newly acquired deterministic, but not probabilistic (Schvaneveldt & Gomez, 1998), sequence structure was observed in individuals with amnesia, when assessed using an indirect test, but not on a task that required the individuals with amnesia to reproduce (generate) the trained event sequence (Vandenberghe et al., 2006). Vandenberghe et al. (2006) also reported that both the individuals with amnesia and control participants were at chance when asked to differentiate between old (i.e., trained) and new (i.e., lure) short sequence based retrieval cues in the context of a recognition memory test.

The use of event sequences based on deterministic, second-order regularities encourages the integration of predictive associations, excludes the possibility of using memory for only one item in a subsequent recognition test for sequence memory, and provides a parallel with prior studies in humans without neurological damage and lesion models of sequence learning involving model organisms (Albouy et al., 2006; Ergorul & Eichenbaum, 2006; Fortin et al., 2002; Rieckmann et al., 2010; Rosenthal et al., 2016; Schendan et al., 2003). Hippocampal activity in functional neuroimaging has been observed when learning second-order regularities, independently of whether learning is incidental or intentional (Schendan et al., 2003) or masked from visual awareness (Rosenthal et al., 2016). Together, these data do not align with the proposition that implicit sequence learning can be independent from declarative memory mechanisms centered on MTL memory system (P. J. Reber & Squire, 1998), but are consistent with the theoretical view that the hippocampus supports the formation and use of stimulus configurations and relations (N. J. Cohen & Eichenbaum, 1993; Rudy & Sutherland, 1995). In addition, most prior studies were based on standard visuomotor implementations of the SRT task, where participants learned sequence-specific information about associations between visible spatial locations, gained general information about mappings between the stimulus and a manual response, and improved perceptual and motor fluency. However, motor learning is not a necessary component of sequence learning (Rosenthal & Soto, 2016; Rosenthal et al., 2009, 2010, 2013; Vakil et al., 2017), and learning under conditions that do not require motor responses may reduce the potential confounds associated with nonspecific perceptual-motor learning (Rosenthal et al., 2010, 2013, 2016; Vakil et al., 2017), which may be important given that stimulus binding can be compromised in amnesia (Race et al., 2019). Therefore, we elected to present event sequences based on a second-order conditional rule in the context of a perceptual sequence learning task that did not require manual responses (Rosenthal et al., 2009, 2013, 2016).

Early biologically plausible models suggested that CA3 supports the association of stimuli across time delays (Rodriguez & Levy, 2001) and can disambiguate sequences (Levy, 1996). However, in a more recent computational network model by Schapiro et al. (2017), known properties of hippocampal projections and subfields were instantiated to address the computational trade-off between memorizing individual experiences rapidly and extracting statistical regularities across experiences. In the model, CA3 and the dentate gyrus (DG) form part of a trisynaptic pathway to the cornu ammonis 1 (CA1) that supports the learning of individual episodes, whereas the monosynaptic pathway (connecting entorhinal cortex directly to CA1), not involving CA3, supports the learning of statistical regularities. Evidence of preserved sequence learning following CA3 damage would suggest that the monosynaptic pathway is sufficient to support the learning of deterministic regularities. In line with the model by Schapiro et al. (2017), a recent case study, based on an individual with selective bilateral damage to the DG, demonstrated that performance was intact on a statistical learning task, which required the rapid generalization and extraction of commonalities shared across multiple auditory events, despite a deficit in the ability to pattern separate (i.e., transform overlapping representations into distinct ones) (Wang et al., 2023).

Experimental evidence, however, suggests that human CA3 may have causal relevance for learning regularities. Engagement of CA3 in binding together disparate pieces of information (i.e., associative learning) can occur during spatial tasks that require the integration of complex environmental cues (Leutgeb et al., 2007; Nakazawa et al., 2002). Furthermore, a recent imaging study indicated that the excitability of rodent CA3 pyramidal cells supported linking temporally discontinuous events, which is a key feature of learning second-order regularities (Eom et al., 2023). Extensive recurrent axon collaterals and a high convergence of inputs from the DG provide CA3 with the capacity to support spatial information processing and consolidation (Diamantaki et al., 2018; Gilbert & Brushfield, 2009; Lin et al., 2021; McHugh et al., 2007; J. O’Keefe & Dostrovsky, 1971; O’Keefe & Nadel, 1978). Thus, the learning of second-order regularities with a spatial component would arguably engage the processing capabilities of CA3 to encode and retrieve the precise order of locations or contexts (Lee et al., 2004). By inference, this specialization may lead to differential contributions to spatial versus non-spatial configurations of perceptual sequence learning. However, early studies also demonstrated that the hippocampus plays a key role in both spatial and non-spatial memory (Cave & Squire, 1991). Indeed, recurrent circuitry in CA3 supports associating one item or event with the next, even when these events are temporal or do not involve spatial locations (Gilbert & Kesner, 2003; Kesner, 2007; Kumaran & McClelland, 2012; MacDonald et al., 2013; Rolls, 1996), and more generally, the integration and representation of multi-dimensional information (Kesner et al., 2004). CA3 can additionally support the integration of contextual cues even without a spatial component, such as an object with a particular outcome or event (Kesner, 2007; Lee & Kesner, 2004; Nakashiba et al., 2008), and both dorsal and ventral CA3 support spatial and non-spatial object recognition memory, albeit with preferential tuning to spatial information (Beer et al., 2013, 2014).

On the grounds that the contribution of CA3 to spatial and non-spatial information processing remains an empirical question, we examined how CA3 damage affected learning deterministic, second-order regularities when presented in the context of two configurations: a spatial perceptual sequence learning task (Experiment 1: the event sequence appeared at one of four fixed spatial locations) and a non-spatial perceptual sequence learning task (Experiment 2: the event sequence appeared at a single fixed central location). If spatial and non-spatial perceptual sequence learning converged in their dependence on computations supported by CA3, including pattern separation and completion (i.e., remembering in response to partial or degraded elements of a stored pattern), temporal association, and sequence encoding (MacDonald et al., 2011), both configurations of the sequence learning task would be likely to be affected by CA3 damage. However, it is also important to note that the dichotomy between spatial and non-spatial tasks is an oversimplification, because each task does not necessarily map directly onto uniquely dichotomized processes. To determine the extent to which any deficits in perceptual sequence learning were secondary to impairments in item memory, two further independent experiments examined memory for novel single-items presented either in a spatial (Experiment 3) or in a non-spatial (Experiment 4) configuration. In all four experiments, memory for newly acquired sequence knowledge was tested on a recognition memory test that followed the initial learning phase. On the basis that recognition memory of unitized single-items can be hippocampal-independent (Bird, 2017), combined with the neuropsychological profile of the individuals with amnesia that was established previously on standardized recognition memory tests (Miller et al., 2017, 2020), the individuals with amnesia were predicted to be able to recognize non-spatial novel items.

In summary, lesion studies in humans have found that medial temporal lobe pathology impairs the implicit learning and recognition of second-order regularities (Curran, 1997; Vandenberghe et al., 2006). Notably, the results from prior studies included individuals with damage that extended beyond both hippocampi. However, it is well established that hippocampal function and anatomy varies by subfield, with each subfield making differential contributions to support integrated (links between memory traces) or differentiated memories (Cowell et al., 2010; Horner et al., 2015; Kumaran & McClelland, 2012; O’Reilly et al., 2014; Schapiro et al., 2017). Accordingly, the current study was based on individuals with episodic amnesia, secondary to bilateral and selective hippocampal damage in a single subregion, human CA3 (Figure 1). All participants underwent four independent experiments: Experiment 1: spatial perceptual sequence learning and a spatial recognition memory test; Experiment 2: non-spatial perceptual sequence learning and a non-spatial recognition memory test; Experiment 3: spatial item learning and a spatial item recognition memory test; and Experiment 4: non-spatial item learning and a non-spatial item recognition memory test (Figure 2). Experiments 1 and 2 involved attending to a repeating visual event sequence under conditions of incidental learning (Rosenthal & Soto, 2016; Rosenthal et al., 2009, 2013), where learning to recognize serial associations between events required knowledge about the order in which non-adjacent events, separated in space and time, unfolded.

**Figure 2.**
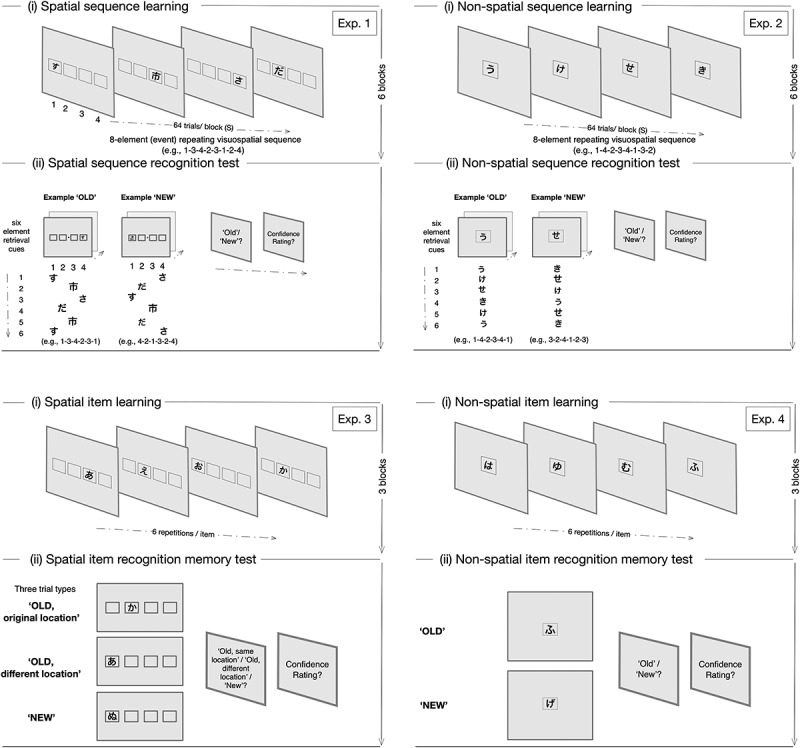
Experimental procedure and stimuli. Each of the four experiments was comprised of a learning and test phase separated by a five-minute delay interval. During the learning phase of all four experiments, participants performed a secondary vigilance task, which involved maintaining an internal cumulative count of bold font-weighted Japanese Hiragana characters presented on each block of trials to facilitate sustained attention to these memoranda. Feedback on performance was provided at the end of each block of trials. At test, the presentation of each retrieval cue was followed by a prompt to endorse each cue as either ‘old’ or ‘new’ (for the exception, see exp. 3), followed by a prompt to rate the confidence associated with each response on a six-point scale (if ‘old’ assign a value ranging in confidence between 1 [certain] and 3 [least certain], or, if ‘new,’ assign a value between 4 [least certain] and 6 [certain]).

## Methods

### Participants

All participants were fluent, native English speakers. Informed written consent was obtained from the participants for all procedures and for consent to publish, in accordance the principles expressed in the Declaration of Helsinki and with the terms of approval granted by the local research ethics committee.

### Group with amnesia

Nine participants with chronic amnesia were recruited (all male, one left-handed). Chronic amnesia was associated with bilateral hippocampal damage, secondary to a single etiology, leucine-rich glioma-inactivated-1 limbic encephalitis (LGI1-LE) (Dalmau & Rosenfeld, 2014; Irani et al., 2010, 2013; Miller et al., 2017). All participants were clinically stable and free-from seizures at the time of testing (median = 4 years post-onset, range = 7), as assessed by their consultant neurologist. The mean age of the participants was 54.4 years (s.e.m. = 4.6). The participants were a subset recruited from a larger group of individuals with amnesia who have been previously reported (Miller et al., 2017, 2020). As reported in the larger group of individuals with amnesia, bilateral and selective damage to the hippocampal CA3 subregion was observed in all of the individuals with amnesia (Figure 1). See below for additional details and our prior work reporting the results from anatomical neuroimaging of these participants (McCormick et al., 2016a, 2016b, 2018; Miller et al., 2017, 2020; Spanò et al., 2020; Spanò et al., 2020).

The participants were otherwise self-reported as healthy and had no evidence of secondary gain or active psychopathology at the time of testing. In contrast to studies on amnesia in individuals with chronic conditions that are associated with seizures and hippocampal sclerosis (Kapur & Prevett, 2003), it follows from the known timing, discrete onset, and monophasic progression of autoimmune encephalitis that hippocampal function was intact when forming memories before the disease onset.

### Control group

Participants in the cognitive behavioral experiments control group were recruited from the community and approximated the ages and years of education of the participants in the group with amnesia. A total of 18 control participants were recruited (mean age = 55.0, s.e.m. = 2.59). There were no significant differences in the age and duration of education between the two groups. Control group participants were also screened to ensure no known history of psychiatric, cognitive, or neurological illness, and were not undergoing a course of psychoactive medications.

### Behavioural experiments 1–4

All participants in the group with amnesia and behavioral control group underwent four independent behavioral experiments (Figure 2). Participants were tested individually, and all were unfamiliar with the Japanese Hiragana character set, which formed the basis for the memoranda used in the four experiments. The four experiments were administered in one experimental session that lasted ∼1 h 30 min. The order of the four experiments was counterbalanced across the participants. Testing was performed on a computer that was configured to a screen resolution of 1024 × 769 pixels and positioned at a 75 cm viewing distance. Each phase of the four experiments was prefaced by written on-screen instructions that were first read by each participant and then discussed with an experimenter to ensure task comprehension. Learning and recognition memory phases in all four experiments were separated by an interval of approximately 5 min and rest breaks were provided after each block of trials. Participants were given practice blocks after reading each set of instructions to ensure task comprehension. All of the behavioral tasks were implemented using E-Prime 2.0 Pro (Psychology Software Tools, Pittsburgh, PA, USA). In all four experiments, the allocation of response keys to outcomes was randomized across the participants.

## Experiment 1: spatial perceptual sequence learning and spatial recognition memory

### Design and materials

Two basic sequences of targets were generated and were based on a second-order conditional (SOC) rule, whereby each event was determined on the basis of the two preceding events (J. Reed & Johnson, 1994). A single set four different characters from the Japanese Hiragana character set were chosen and arranged in two different fixed serial orders to yield two unique eight-event/element second-order conditional sequences (Figure 2). To elaborate further with a worked example, each SOC sequence was based on four Hiragana characters – for the purposes of this worked example, 1, 2, 3, 4 are used here in place of the four Hiragana characters that were presented at screen locations 1–4 – that were arranged into a serial order to generate an eight-event sequence (e.g., 1-3-4-2-3-1-2-4). The second-order property refers to the deterministic relationship in which every upcoming event is fully predicted by the two events that immediately precede it – in the example, ‘4’ follows ’1–3,’ “2” follows “3–4,” “3” follows “4–2,” “1” follows “2–3,” “2” follows “3–1,” and “4” follows ’1–2.’ Such short-range and longer-range dependencies can be acquired by an efficient learning system (Shanks et al., 2006). All eight-events were presented in full before being repeated. The two SOC sequences did not share any SOC units. The length of the eight-event sequence was chosen to minimize fatigue while still testing the capacity to learn a deterministic sequence with second-order conditional properties, and has been shown to be associated with activity in the hippocampus in prior fMRI studies (Albouy et al., 2008; Schendan et al., 2003).

The spatial sequence was presented at four fixed equidistant locations demarked by black squares, aligned with bilateral symmetry along the horizontal meridian of the computer screen and presented against a white background (Figure 2). Each block of the learning phase comprised 64 critical trials (i.e., eight repetitions of the eight-event target sequence). Six training blocks were administered during the learning phase. The two target sequences were counterbalanced across participants, such that each participant was trained on only one of the two basic sequences and the unseen sequence served as the basis for lure retrieval cues on the later, recognition memory test.

Attention to the event sequence was facilitated by presenting each Hiragana character in one of two font-weights – a standard font-weight character and a bold font-weight character (BFWC) – as part of an adapted secondary vigilance task (Rosenthal et al., 2009, 2010, 2013, 2016). BFWCs were presented between 18% and 36% of trials; the order of the BFWCs was random within a block of trials and set at a proportion designed to enable participants to perform at ceiling on the vigilance task. At the end of each block of the learning phase trials, an onscreen prompt appeared asking each participant to report the value for the cumulative count, so that feedback could be provided on their accuracy (see below).

The recognition memory test followed the learning phase and comprised eight trained (old) and eight new six-element sequences used as the retrieval cues (i.e., based on a sequence that was not from the learning phase, but with the same second-order conditional property (i.e., lures)). Eight sequences (starting from each ordinal position of the eight-element SOC sequence for six consecutive locations) were generated from trained SOC and eight were generated from new, lure SOC sequence. Screen locations of the black squares were presented in the same way as in the learning phase. The order of all retrieval cues within a block was random.

### Procedure

Each trial of the spatial perceptual sequence learning task comprised a single Hiragana character presented for 1200 ms (Figure 2, Exp. 1). Participants were asked to fixate on a small circle (0.3 cm diameter) positioned midway between screen locations 2 and 3 (as read from left to right) throughout each block of trials to minimize eye movements, attend to all characters (i.e., not just the bold characters), and maintain an internal cumulative count of the number of BFWCs presented during each block of the learning phase, within ± 5% accuracy of the actual number. At the end of each block of trials, an onscreen prompt appeared asking each participant to report a value for the cumulative count, so that written onscreen feedback could be provided on their accuracy. If the participant’s cumulative value was within 5% of the actual score, the participant was informed that their count was accurate and then asked to continue with their good performance. If the count had an error rate of 5% or greater, then the percentage error underestimation or overestimation was displayed on the screen as feedback and followed by the instruction to improve the counting accuracy on the next block of trials. The accuracy criterion was the same as that applied in previous studies on perceptual sequence learning (Rosenthal et al., 2009, 2013, 2016), and that used in all of the other experiments reported here. Each block lasted approximately 2 min in duration. Participants were given a practice session comprised of six trials to enable them to distinguish between normal font-weight and bold font-weight Hiragana characters.

The recognition memory test for the spatial event sequence followed the spatial perceptual sequence learning task (Figure 2, Exp. 1). On each recognition test trial, a six-element sequence was presented as the retrieval cue, with the parameters for presentation the same as the study phase. A prompt then appeared asking the participant to decide whether the sequence was ‘old/seen’ or ‘new/unseen,’ with respect to the learning phase. Participants were asked to press letter ‘A’ on a keyboard for ‘old’ and the letter ‘S’ on the keyboard for ‘new.’ A 6-point scale was then used to determine the confidence associated with each response. If the participant selected ‘old,’ then the instruction prompted the participant to select one of three numbers corresponding to the following three ratings: (1) I’m certain that this sequence was part of the learning phase; (2) I’m fairly certain that this sequence was part of the learning phase; and (3) I believe that this sequence was part of the learning phase. If the participant selected ‘new,’ then the following three rating options appeared: (4) I believe that this sequence was not part of the learning phase; (5) I’m fairly certain that this sequence was not part of the learning phase; and (6) I’m certain that this sequence was not part of the learning phase.

## Experiment 2: non-spatial perceptual sequence learning and non-spatial recognition memory

### Design and materials

Two basic eight-element/event sequences of targets were generated that followed the SOC rule. The serial order of these two sequences was different from the sequences presented in experiment 1, and each had a different serial order and was based on a single set of four different Japanese Hiragana characters presented at a central location on the computer screen. The four Hiragana characters were different to those used in experiment 1. The two basic sequences were counterbalanced across participants. Each block of the learning phase comprised 64 trials (i.e., eight repetitions of the eight-element target SOC sequence). Six training blocks were administered. Attention to the sequence of visual targets was facilitated by administering the vigilance task. Apart from the presentation of the four Hiragana character-based eight-element sequence at a single central location, all other design parameters of the vigilance task were the same as those implemented in experiment 1. The recognition memory test for the non-spatial sequence followed the learning phase and was comprised of eight trained (old) and eight untrained (new, lure) six-element sequences used as the retrieval cues, with respect to the learning phase. The order of all retrieval cues within a block was random.

### Procedure

Each trial comprised a single Japanese Hiragana character presented for 1200 ms at the center of a fixed location demarked by a single square positioned at the center of the computer screen (Figure 2, Exp. 2). Participants were asked to fixate on the central square throughout each block of the learning phase and maintain a cumulative count of the number of BFWCs presented during each block of the learning phase trials. At the end of each block of trials, an onscreen prompt appeared asking the participants to report the value for the cumulative count, so that feedback could be provided on their accuracy in the same manner as applied in experiment 1.

The recognition memory test followed the learning phase (Figure 2, Exp. 2). On each trial, a six-element sequence was presented at a single central location defined by a black square, with the presentation parameters maintained from the learning phase. A prompt appeared after the offset of each six-element Hiragana character-based sequence asking the participant to decide whether the sequence was ‘old/seen’ or ‘new/unseen,’ with respect to the learning phase. Participants were asked to press the letter ‘A’ on the keyboard for old and press the letter ‘S’ on the keyboard for new. A 6-point scale then followed to determine the confidence associated with each response, in the same manner as described in experiment 1.

## Experiment 3: spatial item learning and spatial item recognition memory

### Design and materials

The target memoranda comprised single Japanese Hiragana characters presented in one of two font-weights at one of four fixed screen locations, demarked by a black square outlined against a white background. The dimensions and locations of the array of four black squares positioned on white background were implemented in the same way as in experiment 1 (Figure 2, Exp. 3). Three blocks of trials were presented during the learning phase and were comprised of 32 individual Hiragana characters presented twice and in a random order, with the constraint that a character was not repeated immediately after an initial presentation.

The recognition test for the location of single items comprised 64 trials. Half of the Hiragana characters were ‘old’ and half were ‘new’ (i.e., the character was not presented in the learning phase). ‘Old’ Hiragana characters were subdivided into two equal trial types: ‘old, original location’ (i.e., a previously presented character appeared in same location as the learning phase) and ‘old, different location’ (i.e., a previously presented character appeared in one of the three other ‘different’ locations with respect to the learning phase). The order of all retrieval cues within a block was random. The assignment of old and new Hiragana characters was counterbalanced across participants. All of the individual characters were unique to the experiment and did not overlap with the other three experiments for each participant.

### Procedure

On each trial of the learning phase, a single, Hiragana character was presented for 1200 ms at one of four fixed locations arranged along the horizontal meridian of the computer screen (Figure 2, Exp. 3). Participants fixated on a circle (0.3 cm diameter) positioned midway between squares 2 and 3 (as read from left to right) throughout each block of trials to minimize eye movements, attend to the spatial location of each character (i.e., not just the bold characters), and maintain an internal cumulative count of the number of BFWCs, as part of the vigilance task. Feedback was provided in accordance with the criteria and manner described for experiments 1 and 2. Participants were informed that the order in which the Hiragana characters appeared would not be tested, only the Hiragana character and its location would need to be remembered.

Each participant then performed a recognition memory test (Figure 2, Exp. 3). On each trial, a single Hiragana character was presented at one of four fixed locations, as in the learning phase. The offset of each retrieval cue was followed by a prompt to decide if the individual character was presented in the learning phase or if the character was a novel Japanese Hiragana character (i.e., ‘new’), with respect to the learning phase. If the character was endorsed as ‘old’, the participant was asked to indicate if it was located either in the original location (i.e., ‘old, original location’) or in a different location (i.e., ‘old, different location’). Responses to the prompt were entered by pressing either A (‘old, original location’), S (‘old, different location’), or D (‘new’) on the computer keyboard. A 6-point scale was then used to determine the confidence associated with each response. If the participant selected ‘old, original location’ or ‘old, different location,’ then the instruction was to select one of three numbers corresponding to the following three ratings: (1) ‘I’m certain that this character was part of the learning phase’; (2) ‘I’m fairly certain that this character was part of the learning phase’; and (3) ‘I believe that this character was part of the learning phase.’ If the participant selected ‘new,’ then the following three rating options appeared: (4) ‘I believe that this character was not part of the learning phase’; (5) ‘I’m fairly certain that this character was not part of the learning phase.’ and (6) ‘I’m certain that this character was not part of the learning phase.’

## Experiment 4: non-spatial item learning and non-spatial item recognition memory

### Design and materials

The target memoranda comprised single Japanese Hiragana characters presented at a single, central screen location, demarked by a black square outlined against a white background. Each block of the learning phase comprised 32 trials, with each character presented twice on each of three blocks. On the recognition memory test, half of the retrieval cues were ‘old/seen’ (16 Hiragana characters) and half of the retrieval cues were ‘new/not seen’ (16 Hiragana characters), with respect to the learning phase. The order of all retrieval cues within a block was random. As in experiment 3, the assignment of old and new Hiragana characters was counterbalanced across participants and all of the individual characters were unique to the experiment for each participant.

### Procedure

Participants were asked to attend to each Japanese Hiragana character that appeared (1200 ms) in a single, central location (Figure 2, Exp. 4). Participants were also asked to keep track of all characters, maintain central fixation, and keep an internal cumulative count of the number of BFWCs presented within a block of trials, as part of the vigilance task administered in the same manner as described in experiments 1–3. Thus, on this task, participants attended to the identity of each character and were informed that the order in which the characters appeared would not be tested.

Each participant then performed a recognition memory test (Figure 2, Exp. 4). On each trial, a single Hiragana character was presented at a single central location. The offset of each retrieval cue was followed by a prompt to endorse the retrieval cue either as previously presented (i.e., ‘old’) or novel (i.e., ‘new’), with respect to the learning phase. The parameters for presentation and instruction to attend were the same as in the learning phase. Participants were asked to press the letter ‘A’ on the keyboard for old and the letter ‘S’ on the keyboard for new. A 6-point scale was then used to determine the confidence associated with each response in the same way as described in experiments 1–2.

### 7.0-Tesla anatomical MRI-based neuroimaging

All participants in the group with amnesia previously underwent anatomical neuroimaging, performed using a 7.0-Tesla whole-body MR scanner (Achieva, Philips), based at the Sir Peter Mansfield Magnetic Resonance Centre, School of Physics and Astronomy, University of Nottingham, operated with a volume-transmit 32-element receive whole-head coil array (Nova Medical, Inc, Wilmington, MA, U.S.A.). Information obtained from the 7.0-Tesla MRI anatomical neuroimaging concerning the nature of the bilateral and selective CA3 lesions in the individuals who comprised the amnesic group has been reported in prior papers (McCormick et al., 2016a, 2016b, 2017, 2018; Miller et al., 2017, 2020; Spanò, Pizzamiglio, et al., 2020; Spanò, Weber, et al., 2020).

Briefly, two main anatomical sequences were acquired to allow different measures. First, whole-brain voxel-by-voxel based morphometry (VBM) and diffeomorphic anatomical registration using an exponentiated Lie algebra (DARTEL) registration method (Ashburner & Friston, 2009). These analyses were performed on three-dimensional whole-brain T_1_-weighted magnetization-prepared rapid acquisition gradient-echo images acquired at 0.6 mm^3^ isotropic spatial resolution, along with non-prepared 3-D images to correct T_1_-weighted images for B_0_ intensity field bias, with the following parameters: 176 slices; resolution = 256 × 256; voxel size = 1 mm × 1 mm × 1 mm; time repetition = 1900 ms; time echo = 2.2 ms; flip angle = 9°. These sagittal T_1_-weighted images provided information on global brain morphology, which enabled us to derive intracranial volume (Mathalon et al., 1993; Nordenskjold et al., 2013). The automated VBM analysis was performed using SPM12 (Statistical Parametric Mapping, Wellcome Trust Centre, London, UK; www.fil.ion.ac.uk/spm). Gray matter volumes from participants in the amnesic and neuroimaging control groups were contrasted using a two-sample *t*-test and thresholded at *p* < 0.05, with family-wise error correction and a cluster extent of 50 voxels. Total intracranial volumes were included in the model as a covariate of no interest. Total intracranial volumes were derived by applying the sequence of unified segmentation, as implemented in SPM12 (Malone et al., 2015), to the T_1_-weighted 7.0-Tesla images of each participant in order to normalize for inter-participant variation and premorbid head size. Second, bilateral quantitative 3-D morphometry of the cornu ammonis (CA) subfields 1–3, dentate gyrus, and subiculum (SUB) was performed on hippocampal images (RF-refocusing sequence optimized for heavy contrast, which provided a three-dimensional T_2_-weighted fast spin-echo partial volume focused on the hippocampi and acquired at 0.39 × 0.39 × 1.0 mm^3^ spatial resolution), acquired as 52 contiguous oblique coronal sections (perpendicular to hippocampal axis), providing coverage of both hippocampi. For additional detail on the analysis pipelines, please see Miller et al. (2017) and Miller et al. (2020).

### Neuropsychological assessment

In order to obtain a detailed neurocognitive profile on each individual with amnesia, all participants underwent an extensive neuropsychological assessment using a battery of standardized neuropsychological tests that assessed the following domains: intelligence, verbal memory, visual memory, recognition memory, attention, language, executive function, visuomotor skills, and visuoconstruction (Table 1). Results from the individuals are a subset of a main group of individuals with amnesia that have been reported previously (Miller et al., 2017, 2020). Each participant was screened for deficits beyond episodic memory because converging evidence indicates that the hippocampus has a broader role in cognition, in areas such as spatial cognition, attention, and perception (Aly et al., 2013; Race et al., 2011; Shohamy & Turk-Browne, 2013; Vargha-Khadem et al., 1997).

**Table 1.** Summary of the neuropsychological screening. Domain scores are based on neuropsychological subtests that are described in our previous studies reporting the full set of participants from which current subset of participants were recruited (Miller et al., 2020). The results include all the individuals with amnesia that took part in the four behavioral experiments reported here.

Neuropsychological	n	ave. *z*-score	S.E.M.	*t*	*d.f.*	p-value
Domain						
Intelligence	9	0.87	0.24	3.56	8	<0.007*
Verbal memory	9	−0.30	0.28	−1.09	8	0.309
Visual memory	9	−0.28	0.20	−1.40	8	0.200
Recognition Memory	9	0.11	0.24	0.44	8	0.675
Attention	8	0.04	0.23	0.16	7	0.881
Executive Function	9	0.60	0.18	3.36	8	0.010*
Language	9	0.65	0.28	2.34	8	0.047*
Visuomotor skills	9	0.14	0.12	1.21	8	0.262
Visuoconstructive skills	9	−0.27	0.59	−0.46	8	0.661

*Group with individuals with amnesia statistically different from normative data, *p* < 0.05, two-tailed one-sample *t*-test; n.=number of patients tested; ave. *z*-score=average *z*-score; s.e.m.=standard error of the mean; d.f., degrees of freedom. Notably, delayed verbal recall performance (which contributed to the verbal memory domain; *n* = 9, ave. *z*-score = −0.57, s.e.m. = 0.32, *t*_(8)_ = −1.80, *p* = 0.109) and delayed visual recall from normative data (comprised of Rey Delayed Recall) (ave. *z* = −0.28, s.e.m. = 0.23, *t*_(8)_ = −1.23, *p* = 0.254) were not significantly different from normative. Delayed verbal recall was comprised of Logical Memory II, Logical Memory II themes and Word Lists II (WMS-III) and People Recall Test.

### Assessment of autobiographical episodic amnesia

Episodic (internal detail) and context-independent (semantic, external detail) autobiographical memory in the group with amnesia and a separate autobiographical memory control group were assessed on the full protocol of the Autobiographical Interview (AI) (Levine et al., 2002; Simpson et al., 2023). The data from the current subset of individuals with amnesia have been reported elsewhere as part of a larger main group of individuals with amnesia (Miller et al., 2017, 2020). Participants in the group with amnesia and AI control group were sampled at identical intervals. Briefly, for each sampled event, verbal prompts were used to facilitate the recovery of perceptual, spatial, and mental state details related to temporally specific recent (within the past year) and remote (extending to ∼60 years) event memories. Results from the current subset group of individuals who underwent the four experiments and the AI control group are reported below. The monophasic nature of LGI1-LE provides a foundation on which to infer that the recent and remote retrograde memories were acquired prior to the illness and represent a measure of hippocampal-mediated retrieval mechanisms.

## Statistical tests

Mixed-model omnibus factorial ANOVAs were used to analyze between-group mean differences for hippocampal subfield volumes and the behavioral results from experiments 1–4. Mauchly’s test was used to assess the assumption of sphericity. Modifications to the degrees of freedom were applied when sphericity was violated so that a valid *F*-statistic could be obtained. Greenhouse-Geisser correction was applied if estimated epsilon (ε) was less than 0.75, whereas Huynh-Feldt correction was applied if the estimated ε exceeded 0.75. Main effects and interaction terms were evaluated using a two-tailed alpha criterion set at *p* < 0.05 to reject the null hypothesis. Follow-up contrasts were used to assess significant main effects and interaction terms, with adjustments to alpha criterion for multiple comparisons based on Holm-Bonferroni correction.

## Results

### Experiment 1: spatial perceptual sequence learning and recognition memory

#### Vigilance task

In both groups, the error rate on vigilance task across the six blocks of trials was less than 5%. These results indicate that the participants in both groups were able to attend consistently to the visuospatial sequence and could reliably differentiate BFWCs from standard font-weight Hiragana characters.

#### Recognition test for the spatial event sequence

**Figure 3.**
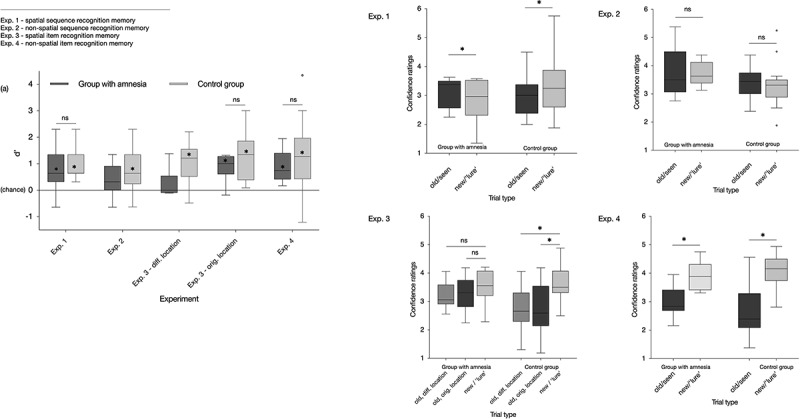
Results from the recognition tests administered after learning a sequence of second-order regularities presented in a spatial (exp. 1) or non-spatial (exp. 2) configuration or single items presented in a spatial (exp. 3) or non-spatial (exp. 4) configuration. Left panel (a): In each of the four experiments, sensitivity to the difference between old and new retrieval cues was calculated using signal detection theory-based d’ scores (chance = 0). Darker gray Tukey box and whisker plots correspond to d’ scores associated with the group of individuals with amnesia, whereas lighter gray Tukey box and whisker plots correspond to the d’ scores associated with the control group. The plots for experiment 3 depict the d’ scores obtained for ‘old, original location’ (i.e., a previously presented Hiragana character that appeared in same location as the learning phase) and ‘old, different location’ (i.e., a previously presented Hiragana character that appeared in one of the three other ‘different’ locations with respect to the learning phase) when presented in an experimental context also presenting ‘new’ items (a Hiragana character that had not appeared during learning phase). Right panel: subjective ratings were based on observers’ confidence associated with each old-new response (if ‘old’/seen, assign a value ranging in confidence between 1 [certain] and 3 [least certain], or, if ‘new’/lure, assign a value between 4 [least certain] and 6 [certain]). * d’ > chance/significant difference between old and new subjective ratings, at *p* < 0.05. ns, contrast not significant at *p* < 0.05.

Recognition memory performance for the spatial event sequence is depicted in Figure 3. In the group comprised of individuals with amnesia, a signal detection theory-based objective measure of sensitivity revealed significant recognition memory for the second-order regularities presented during the learning phase (*d*’ = 0.85, *t*_(8)_ = 2.95, *p* = 0.018, Cohen’s d = 0.984; Figure 3a). Similarly, in the control group, significant recognition memory was evident on the objective measure of sensitivity (*d*’ = 1.02, *t*_(17)_ = 7.00, *p* < 0.001, Cohen’s d = 1.65; Figure 3a). The between-group difference in the above chance d’ scores was not significant (*t*_(25)_=-0.58, *p* = 0.556, Cohen’s d = 0.24). In addition, recognition memory was assessed by conducting a contrast based on confidence ratings associated with all old and all new cues (retrieval cues) (Chong et al., 2014; Rosenthal et al., 2010, 2016, 2018). Accordingly, a mixed-model ANOVA was conducted on the mean confidence ratings with group (amnesic, control) as a between-subjects factor and confidence rating type (old, new) as a within-subject factor (Figure 3, Exp. 1). Degrees of freedom were not adjusted because Mauchly’s test revealed that sphericity was not violated. The main effect of group (*F*_(1,25)_ = 0.903, *p* = 0.351, η^2^_p_ = 0.035) and the interaction between group and confidence rating type (*F*_(1,25)_ = 0.55, *p* = 0.467, η^2^_p_ = 0.021) were not significant, whereas the main effect of confidence rating type was significant (*F*_(1,25)_ = 5.16, *p* = 0.032, η^2^_p_ = 0.171). Taken together, these results indicate significant recognition memory for the spatial event sequence on the objective sensitivity-based measure and on the subjective confidence-based measure for the individuals with amnesia and the control group.

### Experiment 2: non-spatial sequence learning and recognition memory

#### Vigilance task

Performance on the vigilance task for both groups of participants across the six blocks presented during the learning phase was associated with less than 5% error. Hence, both groups of participants were able to sustain attention to the sequence of Hiragana characters at a level that was sufficient to differentiate BFWCs from standard font-weight characters, at the a priori performance criterion.

#### Recognition test for the non-spatial event sequence

Recognition memory performance for the non-spatial event sequence is depicted in Figure 3. In the group of individuals with amnesia, recognition memory for the non-spatial event sequence was at chance on the signal detection theory-based measure of sensitivity (d’ = 0.37, *t*_(8)_=-1.72, *p* = 0.124, Cohen’s d = 0.572; Figure 3a). In comparison, the performance of the control group revealed significant objective sensitivity on the recognition memory test for the non-spatial event sequence (d’ = 0.80, *t*_(17)_ = 4.62, *p* < 0.001, Cohen’s d = 1.09; Figure 3a). A mixed-model ANOVA was conducted on the mean confidence ratings, with group (amnesic, control) as a between-subjects factor and confidence rating type (old, new) as a within-subject factor. Mauchly’s test of sphericity was not significant. The main effect of group (*F*_(1,25)_ = 3.32, *p* = 0.08, η^2^_p_ = 0.118), main effect of confidence rating type (*F*_(1,25)_ = 0.153, *p* = 0.699, η^2^_p_ = 0.006), and the interaction between group and confidence rating type (*F*_(1,25)_ = 0.013, *p* = 0.909, η^2^_p_ = 0.001) were not significant (Figure 3, Exp. 2). Taken together, these results indicate that the ability to recognize the non-spatial second-order regularities was limited to the objective sensitivity-based measure in the control group, whereas there was null differentiation between old and new retrieval cues on the subjective confidence-based measure in the individuals with amnesia and the control group.

### Experiment 3: spatial item learning and recognition memory

#### Vigilance task

The error rate on the vigilance task was less than 5% in both groups of participants. Hence, both groups of participants were able to attend to the individual Hiragana characters presented at one of the four fixed locations and differentiate BFWCs from standard font-weight Hiragana characters, at a level that met the a priori performance criterion.

#### Spatial item recognition memory

Performance on the test of spatial item recognition memory is depicted in Figure 3. In the group of individuals with amnesia, recognition memory for old Hiragana characters in their original location was significant on the signal detection theory-based measure of sensitivity (d’ = 0.85, s.e.m. = 0.16, *t*_(8)_ = 5.26, *p* < 0.001, Cohen’s d = 1.75), whereas sensitivity to old Hiragana characters in a different location was not significant (d’ = 0.27, s.e.m. = 0.17, *t*_(8)_ = 1.61, *p* = 0.146, Cohen’s d = 0.54; Figure 3a). In the control group, sensitivity on the recognition test for Hiragana characters in their original locations (d’ = 1.27, *t*_(17)_ = 6.416, *p* < 0.001, Cohen’s d = 1.51) and Hiragana characters in different locations (d’ = 1.03, s.e.m. = 0.19, *t*_(17)_ = 5.51, *p* < 0.001, Cohen’s d = 1.30) was significant (Figure 3a). The between-group difference in the above chance d’ scores associated with old Hiragana characters presented in their original locations was not significant (*t*_(25)_=-1.38, *p* = 0.180, Cohen’s d = 0.56). A mixed-model ANOVA conducted on the mean confidence ratings was conducted with group (amnesic, control) as a between-subjects factor and confidence rating type (old, original location, old, different location, and new) as a within-subject factor. Mauchly’s test of sphericity was not significant for confidence rating (χ^2^_(2)_ = 2.63, *p* = 0.268). The main effect of group was not significant (*F*_(1,25)_ = 1.676, *p* = 0.207, η^2^_p_ = 0.063), whereas the main effect of confidence rating type (*F*_(1,25)_ = 16.95, *p* < 0.001, η^2^_p_ = 0.404) and the interaction between group and confidence rating type (*F*_(1,25)_ = 4.64, *p* = 0.014, η^2^_p_ = 0.157) were significant (Figure 3, Exp. 3). Follow-up analyses revealed that the mean confidence ratings in the individuals with amnesia between ‘old, original location’ and ‘new’ items (*t*_(8)_=−1.210, *p* = 0.261, Cohen’s d = 0.40) and between ‘old, different location’ and ‘new items’ (*t*_(8)_=−1.88, *p* = 0.096, Cohen’s d = 0.63) were not significantly different. In the control group, confidence ratings associated with old and new retrieval cues in the control group were significantly different between ‘old, original location’ and ‘new’ items (*t*_(17)_=−5.50, *p* < 0.001, Cohen’s d = 1.30) and between ‘old, different location’ and ‘new’ items (*t*_(17)_=−6.48, *p* < 0.001. Cohen’s d = 1.53) at the threshold corrected for multiple comparisons (Figure 3, Exp. 3).

### Experiment 4: non-spatial item learning and recognition memory

#### Vigilance task

In both groups of participants, the error rate on the vigilance task across the three blocks of trials presented during the learning phase was less than 5% error. Hence, the level of performance met the a priori performance criterion in both groups of participants.

#### Non-spatial item recognition memory

Performance on the test of non-spatial item recognition memory is depicted in Figure 3. Item recognition memory for the individual Hiragana characters studied and tested at a single central location was significant on the objective measure of sensitivity for the individuals with amnesia (d’ = 1.06, *t*_(8)_ = 3.37, *p* = 0.010, Cohen’s d = 1.12) and in the control group (d’ = 1.19, *t*_(17)_ = 3.64, *p* = 0.002, Cohen’s d = 0.86) (Figure 3a). Results from Levene’s Test for equality of variances for the between-group difference was not significant (*p* = 0.44). The between-group difference in the above chance d’ scores was not significant (*t*_(25)_=-0.24, *p* = 0.813, Cohen’s d = 0.10). Mauchly’s test indicated the assumption of sphericity was not violated for the mixed-model ANOVA applied to the confidence ratings associated with old and new non-spatial items, in which group (amnesic, control) was a between-subjects factor and confidence rating type (old, new) was a within-subject factor (Figure 3, Exp. 4). The results revealed that the main effect of group (*F*_(1,25)_ = 0.044, *p* = 0.835, η^2^_p_ = 0.002; Figure 3, Exp. 4) and interaction between group and confidence rating were not significant (*F*_(1,25)_ = 1.92, *p* = 0.178, η^2^_p_ = 0.071), whereas there was a significant main effect of confidence rating (*F*_(1,25)_ = 33.85, *p* < 0.001, η^2^_p_ = 0.575). These results indicate that there was a significant difference between the mean confidence ratings associated with old and new non-spatial Hiragana characters in the group of individuals with amnesia and in the control group, and the difference was not modulated by group. Together, these results demonstrate both groups exhibited significant item recognition memory on the objective and subjective measures.

## Autobiographical interview

Mauchly’s test indicated that the assumption of sphericity was not violated for the mixed-model ANOVA applied to the details acquired on the AI, in which group (amnesic, control) was a between-subjects factor and detail type (internal, external) was a within-subjects factor. The results revealed that the main effect of group (*F*_(1,16)_ = 8.69, *p* = 0.009, η^2^_p_ = 0.351), main effect of detail (*F*_(1,16)_ = 123.02, *p* < 0.001, η^2^_p_ = 0.885), and interaction between group and detail (*F*_(1,16)_ = 14.11, *p* = 0.002, η^2^_p_ = 0.469) were significant. Evaluation of the between-group differences on the AI indicated a significant loss of total internal (episodic) detail in the individuals with amnesia as compared to an AI control group (*t*_(16)_=-3.45, *p* = 0.004, Cohen’s d = 1.58), whereas there was no significant between-group difference in total external (semantic) detail generated over five sampled memories (*t*_(16)_=-0.039, *p* = 0.970, Cohen’s d = 0.02). Results from Levene’s Test for equality of variances for each of the between-group contrasts were non-significant (*p*’s > 0.31). In line with our prior reports (McCormick et al., 2016a, 2016b, 2018; Miller et al., 2017, 2020; Spanò, Pizzamiglio, et al., 2020; Spanò, Weber, et al., 2020), the individuals with amnesia in the current study thus exhibited a selective loss in remembering autobiographic episodic information because the between-group difference in remembering was limited to internal detail.

## Neuropsychological assessment

The neuropsychological profile of the group of individuals with amnesia was consistent with cognitive impairment restricted to the domain of episodic autobiographical memory. Neuropsychological domain scores are based on neuropsychological subtests that have been described in our prior studies, and the results from the re-analyses based on the domain scores for the current subset of individuals with amnesia are summarized in Table 1 (Miller et al., 2020). One sample two-tailed *t*-tests were conducted to test whether each domain score was statistically different from normative data (Table 1). Of note, the domain score based on standardized recognition memory tests was not significantly different from normative data and was based on results from Wechsler Memory Scale – third edition [WMS-III] (Weschler, 1997), Warrington recognition memory test (RMT) (Warrington, 1984), and Doors and People test (D+P) (A. D. Baddeley et al., 1994). Furthermore, results from the individual subtests align with domain score. Delayed verbal recognition memory was significantly above normative data on the RMT for common single-words (*z* = 0.89 ± 0.24; *t*_(8)_ = 3.78, *p* = 0.005), and not significantly different from normative data on the WMS-III Word List (*z*=-0.41 ± 0.57, *t*_(8)_=-0.71, *p* = 0.50) and on the D+P Names (−0.59 ± 0.39, *t*_(8)_=-1.53, *p* = 0.39). Delayed visual recognition was also spared on the RMT for unfamiliar faces (*z*=-0.18 ± 0.41, *t*_(8)_=-0.74, *p* = 0.48) and on D+P Doors (*z* = 0.48 ± 0.22, *t*_(8)_ = 2.23, *p* = 0.057). Evidence that the composite score-based domains of verbal (*z*=-0.30 ± 0.28, *t*_(8)=_-0.309, *p* = 0.84) and visual (*z*=-0.28 ± 0.29, *t*_(13)_=-1.40, *p* = 0.200) recognition memory were intact is consistent with the effects of selective damage to regions of the extended hippocampal system (Aronov et al., 2017; MacDonald et al., 2011; Pastalkova et al., 2008); for an exception, see (J. M. Reed & Squire, 1997)). Thus, together with the results from the AI, the neuropsychological profile of the individuals with amnesia is consistent with our prior results that demonstrated the integrity of the extended cortico-hippocampal CA3 circuit was critical for autobiographical episodic recollection, but not for recognition memory of familiar item, face, and object memoranda (McCormick et al., 2016a, 2017, 2018; Miller et al., 2017, 2020; Spanò, Pizzamiglio, et al., 2020; Spanò, Weber, et al., 2020). It is important to note, however, recognition memory for single-items, such as common words or famous faces, which have pre-experimental associations and are tested under conditions that require specific reference to a study list, has been identified with recollection and associated with hippocampal damage (Aly et al., 2010; Bird, 2017; Bird & Burgess, 2008; Trinkler et al., 2009), whereas alternative accounts predict that single-item recognition memory does not depend on recollection and the hippocampus (Brown & Aggleton, 2001; Malmberg et al., 2004).

## 7.0-Tesla anatomical neuroimaging

A three-way mixed-model ANOVA, with one between-subjects variable (group: amnesic, control) and two within-subjects variables (side: left, right; subfield: CA1, cornu ammonis 2 (CA2), CA3, DG, and subiculum (SUB)) was used to test for between-group differences in hippocampal subfield volumes (Figure 1). All subfield volumes were normalized to the total intracranial volumes obtained from the VBM analyses. Mauchly’s test indicated the assumption of sphericity was violated for subfield (χ^2^_(9)_ = 32.07, *p* < 0.001) and for the interaction between subfield and side (χ^2^_(9)_ = 40.01, *p* < 0.001). Degrees of freedom were corrected using Greenhouse-Geisser estimates (subfield, ɛ = 0.526; side x subfield, ɛ = 0.467). Volumes were collapsed across the left and right hippocampi because there was no significant interaction between group (amnesic, control), side (left, right), and subfield (CA1, CA2, CA3, DG, and SUB) (*F*_(1.87,31.14)_ = 0.49, *p* = 0.604, η^2^_p_ = 0.030). Levene’s test for the equality of variances were not significant for the follow-up contrasts (*p’s* > 0.06). Volume loss in CA3 was significant and survived Holm-Bonferroni correction for multiple comparisons (*t*_(16)_=-3.35, *p* = 0.004, Cohen’s *d*=-1.58). By contrast, volume losses in CA1 (*t*_(16)_=-1.88, *p* = 0.079, Cohen’s *d* = 0.89), CA2 (*t*_(16)_=-1.19, *p* = 0.253, Cohen’s *d* = 0.56), DG (*t*_(16_ =-0.71, *p* = 0.491, Cohen’s *d* = 0.33), and SUB (*t*_(16)_=-1.74, *p* = 0.101, Cohen’s *d* = 0.82) were not significant. In summary, quantitative hippocampal subfield volumetry (assessed at 390 µm in-plane resolution) and whole-brain voxel-based morphometry on anatomical MRI images acquired at 7.0-Tesla demonstrated that all participants in the group with amnesia exhibited bilateral and selective hippocampal volume loss in CA3 relative to a neuroimaging control group.

## Discussion

In the current study, we investigated the causal contributions of human CA3 to the implicit learning of complex event sequences presented either in a spatial (experiment 1) or in a non-spatial (experiment 2) configuration. The extent to which these contributions were secondary to an impairment in item memory was addressed by testing recognition memory for single novel items presented either in a spatial (experiment 3) or a non-spatial configuration (experiment 4). Results from the first experiment, where the event sequence was presented and tested at four fixed spatial locations, revealed that damage to CA3 did not affect the ability to recognize trained second-order regularities, as measured in terms of above chance d’ and on the subjective confidence-based measure relative to the control group. By contrast, in experiment 2, recognition memory for the second-order regularities presented at a single location was at chance in the individuals with amnesia, whereas the control group exhibited significant sensitivity on recognition memory for the non-spatial sequence but were unable to differentiate between old and new sequences on the subjective-based measure. In experiments 3 and 4, the individuals with amnesia and the control group exhibited above chance sensitivity in the recognition memory test for single novel items, whereas memory for the location of an item presented in a different location from the learning phase was at chance in the group with amnesia but evident in the control group. Together, the results demonstrate that damage to the human CA3, associated with temporally ungraded autobiographical episodic amnesia, impaired recognition memory for non-spatial but not spatial event sequences. Evidence of intact of recognition memory for novel items suggests that the selective deficit was unlikely to be secondary to a general impairment in item-specific memory. We discuss each of the three main results in turn.

First, intact recognition memory for the spatial event sequence following hippocampal damage does not align with prior results from human and infra-human lesion studies reporting hippocampal dependence when learning to recognize second-order regularities (Curran, 1997; Ergorul & Eichenbaum, 2006; Reber & Squire, 1998; Shanks et al., 2006). Intact sequence learning has been reported in individuals with hippocampal damage, yet evidence of newly acquired sequence knowledge was detected only on an indirect performance-based RT measure (Curran, 1997; Nissen et al., 1989; Reber & Squire, 1994, 1998; Shanks et al., 2006; Vandenberghe et al., 2006), and not on follow-up verbal report nor on a subsequent recognition test (Curran, 1997; Reber & Squire, 1998; Shanks et al., 2006; Vandenberghe et al., 2006). The ability to recognize the event sequence suggests that the knowledge was conscious because the participants were required to reflect on the learning phase, though it remains conceivable that between-group differences would have emerged on explicit tests which required the generation of an event sequence based on the newly learned contingencies (Rosenthal et al., 2009, 2013; Shanks et al., 2006; Vandenberghe et al., 2006). Evidence of intact recognition memory for second-order regularities runs counter to the view that amnesia reflects a deficit in the capacity to bind different objects (i.e., relational memory) (Cohen & Eichenbaum, 1993; Ryan et al., 2000), because explicit recognition of the second-order regularities was dependent on the binding of non-adjacent location and temporal dependencies. In other accounts, however, relational binding between items or items-to-contexts emerges from interactions between brain regions rather than from a single region such as the hippocampus (Schedlbauer & Ekstrom, 2017).

A potential source of the discrepancy between the current results and prior studies of implicit learning involving damage to the hippocampus is the etiology and extent of the neuroanatomical pathology. In the study by Curran (1997), the individuals with amnesia had mixed etiology (Korsakoff’s Syndrome [4], encephalitis [2], anoxia [3], and thalamic infarcts [1]). This was also the case for the studies by Reber and Squire (1994, 1998) (Korsakoff’s Syndrome [2], epilepsy [1], and herpes simplex encephalitis [1], and unknown etiology [1]), Van Tilborg et al. (2011) (Korsakoff’s Syndrome [20]), Shanks et al. (2006) (Korsakoff’s Syndrome [4], encephalitis [4], and unilateral lesions [2], one due to left-sided posterior communicating artery infarct and the other due to a right-sided tumor), and Vandenberghe et al. (2006) (Korsakoff’s Syndrome [4], aneurysm [1], and closed-head trauma [1]). Most of these etiologies lead to diffuse anatomical damage that extends beyond the hippocampal formation (Miller et al., 2017, 2020). Furthermore, the specific details related to the neuroanatomical damage in these prior studies were either not confirmed with anatomical neuroimaging (Curran, 1997; Shanks et al., 2006; Vandenberghe et al., 2006) or reported only in a subset of the individuals with amnesia (Reber & Squire, 1998; Shanks et al., 2006). By contrast, the model of hippocampal damage investigated in the current study was based on a single monophasic etiology, autoimmune limbic encephalitis, that has been shown to be associated with bilateral and selective hippocampal damage (McCormick et al., 2016a, 2016b, 2017, 2018; Miller et al., 2017, 2020; Spanò et al., 2020; Spanò et al., 2020).

Another consideration is that differences in the protocols used to study implicit sequence learning hinder simple cross-study interpretations. Some protocols applied in human lesion studies involved trial-unique event sequences, which is a key feature of episodic memory, or interleave structured and random event sequences in the same manner as many human neuroimaging studies that find hippocampal activity (Curran, 1997). In other reports, as was the case in the current study, the same event sequence was repeatedly presented during the learning phase (Reber & Squire, 1994; Reber & Squire, 1998). Sequence learning has also been investigated under conditions of feedback or rewards attached to order judgments during learning (DeVito & Eichenbaum, 2011). Individuals with primary damage to the bilateral hippocampus have also been found to be impaired when asked to solve spatial and non-spatial configurations of a novel associative task based on incremental reinforcement learning (Kumaran et al., 2007). Other implicit learning paradigms that require the detection of complex patterns in stimuli, such as artificial grammar learning or learning statistical structure to develop expectations that facilitate decision-making (Chalk et al., 2010; Kim et al., 2009; Ordin et al., 2020; Perruchet & Pacton, 2006; Wald & Wolfowitz, 1949), also appear to be supported by the hippocampus (Chun & Phelps, 1999; Schapiro et al., 2014). Notably, however, recent work suggests that learning expectations based on statistical regularities can be hippocampal independent, particularly when damage does not extend beyond the hippocampus, and has been interpreted as a evidence for dissociable declarative and procedural learning system when observed together with episodic amnesia (Rungratsameetaweemana et al., 2019).

It is also important to consider the potential for possible changes in other brain regions following CA3 damage. Evidence from our prior work examining the effects of CA3 damage on measures of resting-state functional connectivity revealed that CA3 damage disrupted functional integration across the MTL subsystem of the default network (Miller et al., 2020). These results point to the limits on defining even selective hippocampal damage solely in terms of discrete, anatomical descriptions, especially given that episodic memory in non-additive graph theoretic models emerges from distributed interactions between brain hub regions (A. M. Schedlbauer & Ekstrom, 2019). Correspondingly, when evaluating the alignment between functional neuroimaging and patient studies, another factor is that implicit learning involves an interconnected network of regions alongside the hippocampus, including the caudate nucleus and superior parietal lobule (Ling et al., 2015), the basal ganglia (right putamen and pallidum), inferior temporal gyrus, and visual areas that include primary visual cortex (Batterink et al., 2019; Rosenthal et al., 2016). By extension, if connectomic diaschisis, whereby functional changes occur in regions that are not directly linked to a damaged area (Carrera & Tononi, 2014), underlies the ability of the individual with amnesia to recognize second-order regularities, it is conceivable that other networked brain regions compensated for the required information processing (Murray et al., 2017). This reconfiguration of regions can continue to alter dynamically in response to changing task demands (Ferbinteanu, 2019). Support for this perspective is available even in the absence of pathology, because different strategies can lead to dissociable neural substrates in tasks such as artificial grammar learning (Batterink et al., 2019; Opitz & Friederici, 2004), modulate network-wide functional connectivity on the SRT task (Bassett et al., 2015), and are associated with discrete event-related potentials on the weather prediction task (Rustemeier et al., 2013).

Second, the results from experiment 2 indicate that damage to human CA3 impaired learning to recognize second-order regularities presented at a single location. Unlike the results from experiment 1, the deficit in learning to recognize the second-order regularities aligns with experimental evidence showing that activity in CA3 has a role in the integration of information (Berron et al., 2016; Grande et al., 2019). This has been observed under conditions without a spatial component (Kesner, 2007; Lee & Kesner, 2004; Nakashiba et al., 2008), and may reflect the CA3 contribution to relevant computations such as pattern separation and completion, temporal association, and sequence encoding (MacDonald et al., 2011). Moreover, the observed deficit is consistent with the potential relevance of learning second-order regularities for understanding the temporal organization of past experience, along dimensions such as the coding of temporal order judgment, serial order, and the distance of events within an episode, which are the types of impairment observed following hippocampal damage in humans and rodent lesion models (Eichenbaum, 2013; Fortin et al., 2002; Milner, 1971; Palombo et al., 2016; Shimamura et al., 1990; Squire et al., 1981). Finally, more widescale anatomical damage to the human hippocampus can affect behavioral tasks that do not have an explicit spatial component, such as learning novel associations between word pairs (Eichenbaum, 2004; Squire et al., 2004). Notably, however, when the deficit is reconsidered in the context intact recognition memory for the spatial sequence in experiment 1, it is not due to a general inability to learn second-order regularities and is inconsistent with what would be predicted from the perspective of experimental work indicating that CA3 exhibits preferential spatial tuning.

As noted earlier, the neural model by Schapiro et al. (2017) reconciles the processes of episodic memory and statistical learning, through a division of labor between the DG-CA3 trisynaptic pathway and the entorhinal-CA1 monosynaptic pathway. Notably, in a modification designed to test the impact of a ‘lesioned’ dentate gyrus-CA3 trisynaptic pathway, the entorhinal-CA1 monosynaptic pathway was sufficient to support the learning of statistical regularities. Hence, unlike the results from learning to recognize the non-spatial sequence, the evidence of intact learning of deterministic regularities in experiment 1 is broadly consistent with the model. However, the model was focussed on the learning process, rather than recognition memory, and it was not aimed at distinguishing the effects of spatial versus non-spatial configurations of the learning environment. Furthermore, the model was not intended to capture the prediction of pairwise sequential, deterministic dependencies that characterize second-order regularities; instead, the model addressed simple and higher-level regularities involving community structure, where the relationships between elements were defined by their temporal proximity and co-occurrence. To develop an a priori foundation on which to address the results from sequence learning under spatial and non-spatial configurations, it will thus also be necessary to incorporate theoretical quantities related to the mechanisms of connectomic diaschisis and specify how cooperation between affected brain regions responds to different learning strategies under different configurations.

On face value, the presentation of events in an overlapping location generated different computational demands relative to the spatial configuration, which were not supported by the residual function. Indeed, spatial organization can facilitate learning and retrieval, even when the specific information sought from memory is not overtly spatial (Gaffan, 1991). Evidence from SRT task indicates that sequence learning can be impaired when stimulus and response sequences are represented by symbols rather than spatial locations (Koch & Hoffman, 2000). Nonetheless, in the current study, the control group exhibited significant recognition memory for the spatial and non-spatial perceptual sequences, and spatial and non-spatial information processing are not necessarily strictly segregated (Wu et al., 2020; Zhang et al., 2022). However, presenting an event sequence in a spatial context could aid the organization of event order and detection of causality, support learning through greater stimulus-response compatibility, and improve how information is represented in working memory, thereby potentially mitigating interference effects (Schwarb & Schumacher, 2010; Yousif et al., 2021; Zacks, 2020). Therefore, the deficit may reflect sensitivity to the context available in the spatial versus non-spatial learning configurations, particularly given that medial temporal lobe damage and diazepam-induced amnesia impairs the ability to use context to support accurate recognition memory (Shanks et al., 2006). Notably, future studies that address the role of context will need to operationalize the construct adequately so that the specific mechanisms at play can be unpacked (S. M. Stark et al., 2018).

Furthermore, on the basis that recognition memory for the second-order regularities was intact in experiment 1, it would be productive to test whether non-spatial sequence knowledge is detectable on an indirect, incidental measure that does not reference memory, because prior studies report evidence of dissociations between direct and indirect measures in the context of studies on sequence and statistical learning (Batterink et al., 2015; Curran, 1997; Hopkins et al., 2004; Rungratsameetaweemana et al., 2019; Vandenberghe et al., 2006). Speculatively, if explicit processing differentially facilitates the learning of a non-spatial event sequence, then reducing dependence on information processing associated with explicit memory might mitigate the observed difference between the group with amnesia and control group. However, it is becoming increasingly challenging to separate functional properties along a division of implicit and explicit information processing (Hassin, 2013; Meyen et al., 2023; Soto et al., 2019). Nonetheless, memories need to be malleable in order to handle changes in utilization and retrieval demands (Roüast & Schönauer, 2023; Sekeres et al., 2018; Winocur et al., 2007, 2010). These features have often been linked with MTL-dependent conscious, episodic information processing in regions that include the hippocampus (Frankland & Bontempi, 2005; O’Reilly & Norman, 2002; Roüast & Schönauer, 2023). Thus, another potential mediating variable to account for the observed deficit in the non-spatial relative spatial sequence configuration is that residual function was insufficient to support adapting the newly acquired sequence knowledge rapidly for the pattern completion demands associated with the partial retrieval cues on the recognition test, particularly given that CA3 also contributes to pattern completion (Guzowski et al., 2004; Knierim & Neunuebel, 2016; Leutgeb & Leutgeb, 2007).

Another consideration is that learning to recognize more than one second-order conditional sequence could be potentially confounded by order-related effects. For example, the serial position in which an experiment was presented may have influenced how the task was approached, and thereby potentially altering the network of regions and computations that supported task performance. It is also conceivable that learning was vulnerable to proactive interference because of the common structural properties between the sequences presented on each experiment. Although evidence from statistical learning indicates that learning more than one set of regularities can diminish the learning of additional regularities (Gebhart et al., 2009; Junge et al., 2007), a recent neuroimaging study demonstrated that multiple concurrent deterministic, second-order regularities can be learned in parallel (Rosenthal et al., 2018), which aligns with behavioral studies showing that sequences can be learned concurrently (Goschke & Bolte, 2012; Wilts & Haider, 2023). Nonetheless, the individuals with amnesia are likely to have been more vulnerable than the control group to cross-talk and other carry over effects, given that hippocampal damage affects how overlapping memory traces are disambiguated and how interference is handled (Dewar et al., 2010; Squire & De de, 2015; Yonelinas et al., 2019). However, order-related effects were likely to have been minimized by the counterbalancing of the order in which the four experiments were presented across the participants. Systematic interference effects would, arguably, have been further minimized by the delays introduced between each learning session, the lack of overlap in the serial order on each sequence, and the unique set of Hiragana characters associated with each trained sequence. There is also no clear a priori mechanism to explain the deficit on non-spatial sequence learning due to an order effect alone because the experiment did not systematically precede or follow the spatial sequence learning task or a particular item task. In future work, a strategy that could mitigate cross-talk when learning each sequence would be to introduce additional contextual cues, such as different environments or changes to the secondary vigilance task between each experiment, on the basis that contextual cueing can have a strong influence on memory and attention (Chun & Jiang, 1998; Manelis & Reder, 2012).

It is important to note that the recognition memory deficit for the non-spatial event sequence is unlikely to be secondary to impairments in cognitive faculties that are necessary for sequence learning, such as attention, working memory, and executive function, because intact performance was observed on these domains when assessed using standardized neuropsychological tests. These results are in line with prior studies from our laboratory and other laboratories on the chronic phase of the LGI1-limbic encephalitis phenotype (Argyropoulos et al., 2019; Butler et al., 2014; Frisch et al., 2013; Malter et al., 2014; McCormick et al., 2016a, 2017, 2018; Miller et al., 2017), whereas more extensive hippocampal damage is often associated with a broader pattern of cognitive deficits (Aly et al., 2013; Hamann & Squire, 1997; Olson et al., 2006). More diffuse MTL damage can also influence visual information processing and perception, presumably because early and later visual areas have direct projections to several putative memory structures in the MTL including the hippocampus (Dalton et al., 2022). For example, MTL damage can impair perceptual processing, such as the perceptual disambiguation of overlapping stimulus representations, in addition to supporting aspects of associative recognition memory (Baxter, 2009, Bussey et al., 2006; but see, Suzuki & Eichenbaum, 2000). By contrast, perceptual information processing was preserved in the individuals with amnesia, at least as measured in terms of their performance on standardized neuropsychological assessment and in their ability to make the type of fine-grained discrimination required on the tests of recognition memory for the novel items.

Third, the results from experiments 3 and 4 revealed that damage to human CA3 did not disrupt item recognition memory for the individual Japanese Hiragana characters. These results are consistent with the absence of dysfunction on the standardized tests of item recognition memory, and consistent with other studies that have reported intact item recognition memory following selective hippocampal damage (A. Baddeley et al., 2001; Mayes et al., 2002). Other laboratories have reported that item recognition memory is supported by MTL regions that include the hippocampus (Gold et al., 2006; Kirchhoff et al., 2000; Otten et al., 2001; Stark & Squire, 2003), and hippocampal damage can have negative impacts on item and source memory for single words (Gold et al., 2006). Furthermore, impaired recognition of abstract patterns has been found following hippocampal damage (Levy et al., 2003), though the deficit may reflect the hippocampal-dependent processing of contextual associations (Bird, 2017). The retrieval of individual Hiragana characters, if perceived as an integrated, single unit, would be unlikely to trigger preexisting conceptual information. Such conditions support hippocampal-independent retrieval and thus preserved item recognition memory following hippocampal damage (Bird, 2017).

Nonetheless, the individuals with amnesia exhibited a deficit when the items were presented in a different location with respect to the learning phase compared to the control group performance, at least over the range of mnemonic indices obtained in the current study. This is perhaps unsurprising if successful item recognition for changes between study and test was dependent on the binding of item to a particular spatial location (i.e., ‘where’ component). These demands were likely to be greater than on the spatial sequence learning task because of the greater number of unique Hiragana characters presented and lower number of presentations to bind each item to a location. Selective hippocampal damage has been shown to disrupt recognition memory, when associated with binding representations between items and items to contextual information (Bird, 2017; Winters et al., 2004), and memory for allocentric spatial information (R. C. O’Keefe & Nadel, 1978). In addition, if the deficit in recognition memory for the location of individual Hiragana characters was dependent on pattern separation-based computations, then damage to CA3 is likely to have been a mediating variable in the observed performance, given the established role of CA3 in pattern separation computations (J. Kim & Yassa, 2013). Furthermore, the demands on pattern separation computations associated with the greater number of lures and targets on the item versus sequence spatial task may be relevant to the observed deficit, because human hippocampal damage impairs the ability to recognize lures related to the studied items (Kirwan et al., 2012). An additional consideration is that, compared with the non-spatial item task, the greater number of individual items presented at study on the spatial item task will have also contributed proactive and retroactive interference, and increased output interference over the course of the longer test list (Criss et al., 2011). It will thus be important to investigate spatial item recognition memory over a broader range of experimental conditions in future work.

## Awareness and learning to recognize event sequences

A core assumption of early standard views of conscious memory is that hippocampal involvement in implicit learning occurs only when there is contamination from explicit memory processes (N. J. Cohen, 1984; N. J. Cohen & Squire, 1980; Moscovitch, 1992), and thus conscious awareness of what has been learned is considered a key feature of hippocampal-dependent memory (Smith & Squire, 2018). Accordingly, intact learning in individuals with amnesia on implicit or non-declarative tasks in the absence of conscious recollection has been a mainstay of evidence to suggest learning occurred without awareness (Clark et al., 2002; N. J. Cohen & Squire, 1980; Hamann & Squire, 1997; Knowlton & Squire, 1996; Knowlton et al., 1994, 1996; Milner et al., 1968; Moscovitch, 1992; Squire & Dede, 2015). However, there are several contentious issues associated with this approach that are beyond the scope of the current discussion, but some key issues include the following points. First, it is inherently circular to infer the status of implicit or non-declarative tasks in terms whether individuals with amnesia exhibit intact or impaired performance (Nadel, 1994). Second, both implicit and explicit learning-based mechanisms operate in parallel and are non-exclusively engaged during the learning of visible event sequences (Batterink et al., 2019; Perruchet et al., 1990; Sanchez & Reber, 2013), and the acquired knowledge can be brought under intentional, conscious control (Wilkinson & Shanks, 2004). Third, a reduced learning rate in a single explicit learning system can capture functional deficits associated with amnesia, without the need to posit implicit and explicit memory systems (Kinder & Shanks, 2001).

Additional concerns are that verbal report or subjective tests are not necessarily representative of newly acquired knowledge, can be unreliable assays of the state of awareness, and are often based on ‘vague criteria’ (Petersson et al., 2012). Very few studies have elected to exclude conscious access to an event sequence by using visual masking both at study and at test (Rosenthal & Soto, 2016; Rosenthal et al., 2010, 2016; Soto et al., 2019). Even when conscious access is excluded (i.e., d’ = 0 on tests of recognition memory and objective assays of awareness), hippocampal activity can be associated with sequence learning, and may reflect involvement in forming spatial and temporal associations between items represented in cortical areas (Eichenbaum, 2000; Rosenthal & Soto, 2016; Rosenthal et al., 2016). Evidence of intact recognition memory supports the notion that the individuals with amnesia possessed awareness of what had been learned, because recognition memory is one of most studied and important examples of explicit and declarative memory (Bird, 2017).

More recent theoretical treatments have focussed on the computational processes supported by specific brain regions such as the hippocampus and characteristics of representations, independently of the state of awareness associated with memories (Hannula & Greene, 2012; Henke, 2010; Kim, 2019; Moscovitch et al., 2016). The hippocampus under these treatments is critical for the binding and representation of flexible, relational binding of items to contexts even without awareness (Cohen & Eichenbaum, 1993; Eichenbaum et al., 1994). Recent evidence consistent with this perspective indicates that early visual areas and the hippocampus are associated with learning second-order regularities that are masked from visual awareness (Rosenthal et al., 2010, 2016). Importantly, it does not necessarily follow that above-chance recognition memory observed in the current study is process-pure (Jacoby, 1991, 1998), where performance is mediated exclusively by conscious access to remembered information (Hannula et al., 2023; Rosenthal & Soto, 2016; Rosenthal et al., 2016; Soto et al., 2019). Therefore, it would be interesting to test how individuals with amnesia and controls would differ when learning is isolated from conscious knowledge of the sequence.

## Conclusions

Individuals with amnesia, secondary to bilateral and selective hippocampal damage in a single subregion, CA3, exhibited significant recognition memory for a spatial event sequence based on second-order regularities, but were at chance on a test of recognition memory for an event sequence presented in a non-spatial reconfiguration. Thus, the effects of human hippocampal CA3 damage did not have a generalized impact on learning to recognize complex event sequences, as observed in prior human and infra-human lesion studies. The pattern of results also did not align with prior evidence of preferential spatial tuning of CA3. The observed deficit was unlikely to be secondary to losses in perception, attention, and working memory or remembering single items because the individuals with damage to CA3 were intact on these domains, at least as assessed in terms of performance on standardized neuropsychological tests. In contrast to the results from the sequence tasks, the deficit in spatial item recognition memory is arguably consistent with prior evidence suggesting preferential spatial tuning of CA3. A further implication of the results is that the processes that support autobiographical episodic memory – which are impaired by CA3 damage – do not readily overlap with learning to recognize serial associations between events separated in space and time. Evidence from functional neuroimaging implicates the hippocampus in the temporal organization of memory and binding contiguous association (Clewett et al., 2019; Eichenbaum, 2013), which underlies our ability to remember when past episodes occurred (Allen et al., 2016; Clewett et al., 2019; Fortin et al., 2004; Ranganath & Hsieh, 2016; Tulving, 2002), as part of a network that includes the hippocampus and prefrontal cortex (Allen et al., 2014; Allen & Fortin, 2013). However, the extent to which these operations are necessarily linked to the deficits in conscious information processing remains an empirical question that will need to be studied. More generally, the results from the current study provide a foundation on which to address the impact of subfield damage on implicit learning, but it remains to be determined how other regions of hippocampal formation (i.e., the DG, CA fields, and subicular complex) contribute to the learning of complex information. This work will need to build on the highly conserved nature of the hippocampal formation across mammalian species, which has enabled the necessity of the hippocampus in implicit learning tasks to be extensively evaluated in model organisms (Allen et al., 2016; Christie & Dalrymple-Alford, 2004; Christie & Hersch, 2004; Ergorul & Eichenbaum, 2006; Fortin et al., 2002; Schwarting, 2009).

## Data Availability

The data used to support the results of this study are not available for open distribution to comply with the restrictions of the local research ethics committee and the terms of consent signed by the human participants. For further details on the restrictions related to data sharing, please email clive.rosenthal@clneuro.ox.ac.uk.
